# Optical Coherence Tomography May Help Distinguish Glaucoma from Suprasellar Tumor-Associated Optic Disc

**DOI:** 10.1155/2019/3564809

**Published:** 2019-10-29

**Authors:** Michael Mimouni, Hadas Stiebel-Kalish, Irena Serov, Gabriel Chodick, Mohammad Zbedat, Dan D. Gaton

**Affiliations:** ^1^Department of Ophthalmology, Rambam Health Care Campus, Haifa, Israel; ^2^Technion—Israel Institute of Technology, Haifa, Israel; ^3^Department of Ophthalmology, Rabin Medical Center-Beilinson Hospital, Petah Tikva, Israel; ^4^Neuro-Ophthalmology Unit, Department of Ophthalmology, Rabin Medical Center-Beilinson Hospital, Petah Tikva, Israel; ^5^Sackler School of Medicine, Tel Aviv University, Tel Aviv, Israel; ^6^Department of Epidemiology and Preventive Medicine, Tel Aviv University, Tel Aviv, Israel

## Abstract

**Purpose:**

This study aimed to differentiate patients with bilateral disc cupping associated with suprasellar tumor from patients with open-angle glaucoma by analyzing differences in optical coherence tomography (OCT) of the optic nerve.

**Methods:**

In this retrospective cross-sectional study, we collected data from the eyes of 25 patients with suprasellar craniopharyngioma or pituitary macroadenomas (group 1) and 35 patients with primary open-angle glaucoma (POAG) (group 2), seen between 2001 and 2015, all with a visual acuity of ≥20/40, for whom Stratus Time-Domain (TD) optic nerve OCT scans were available. The main outcome measures were the retinal nerve fiber layer (RNFL) thickness, disc area, cup volume, cup/disc ratio, and rim area.

**Results:**

A total of 31 patients met the inclusion criteria and were included in the study: 16 with suprasellar tumors and 15 with POAG. Both groups were similar in terms of gender and age (*P* > 0.05). The glaucoma group had a borderline greater total RNFL thickness (74.2 *μ*m versus 62.8 *μ*m, *P*=0.07), disc area (2.70 mm^2^ versus 2.16 mm^2^, *P*=0.004), and cup volume (0.20 mm^3^ versus 0.08 mm^3^, *P*=0.02). In multivariate, glaucoma was associated with increased total RNFL thickness (OR = 1.116 per *μ*m, *P*=0.008), increased disc area (OR = 2.402 per 100 *μ*m^2^, *P*=0.002), and decreased rim area (OR = 0.272 per 100 *μ*m^2^, *P*=0.011). Of these, the parameter with the greatest AUC was the disc area (AUC = 0.79). Using the Youden index, the optimal cut-off point identified for stratification was a disc area greater than 2.33 *μ*m^2^.

**Conclusions:**

In patients with bilateral disc cupping, a decreased total RNFL thickness and smaller disc area seem to be associated with suprasellar tumors (when compared with open-angle glaucoma). These findings may aid in early diagnosis of cupping from suprasellar tumors, before compressive visual loss occurs.

## 1. Introduction

The clinical hallmark of optic disc cupping can be an insufficient tool to differentiate glaucomatous from other optic neuropathies [1, 2]. Increased cupping can also occur in patients with suprasellar tumors compressing the anterior visual pathway [1–3]. Patients typically develop increased disc cupping before central visual loss occurs and are often first referred to a glaucoma service, where differentiation from other glaucoma patients may prove to be a challenge.

Neuro-ophthalmologists are trained to combine data from the clinical history and multiple optic nerve functions to determine which patient with disc cupping requires neuroimaging. Although there are functional and structural findings that lead to a neuro-ophthalmological examination with neuroimaging [4], these may be overlooked in a busy glaucoma service to which patients with optic disc cupping with visual defects may be referred.

In glaucoma, cupping is associated with optic canal expansion [2]. Indeed, previous studies have reported that increased disc area may be associated with glaucoma [5] and that canal expansion occurs with increasing intraocular pressure [6]. Our goal was to analyze whether canal expansion in glaucoma can be used to differentiate suprasellar tumors from glaucoma using optic nerve head OCT images. We suggest that disc area, a measure which is easily accessible from the OCT scans, can serve as an additional tool to help differentiate patients with compressive suprasellar tumors, who often develop optic disc cupping even before compressive visual loss occurs.

## 2. Methods

The study followed the tenets of the Declaration of Helsinki and was approved by the Institutional Review Board of the Rabin Medical Center.

In this retrospective study, we collected time-domain optical coherence tomography (TD-OCT) variables from the eyes of 25 patients seen from 2001 to 2015 with suprasellar craniopharyngioma or pituitary macroadenomas (group 1) and 35 patients with primary open-angle glaucoma (POAG) (group 2). Patients selected had a visual acuity of ≥20/40 and had complete Stratus Time-Domain optic nerve OCT (Carl Zeiss, Dublin, CA) scans with a signal strength ≥6. In the suprasellar tumor (group 1), patients were excluded if their tumors caused clinical signs of compressive optic atrophy by directly compressing one or both optic nerves. Patients with a severely tilted disc or peripapillary atrophy that could potentially affect OCT measurements were excluded. The POAG group (group 2) consisted of patients previously diagnosed and treated as POAG based on the presence of glaucomatous optic nerve damage (e.g., neuroretinal rim notching or thinning, RNFL defect, and optic disc hemorrhage) with or without associated visual field defects. Excluded were patients with advanced glaucoma defined as near total cupping of the optic nerve with or without severe visual field loss within 10° of fixation, i.e., scotoma encroaching on or splitting fixation [7]. In addition, patients with a best-corrected visual acuity worse than 20/40 in the studied eye or any POAG patients after glaucoma surgery as well as patients with other types of glaucoma were excluded. In addition, patients with a history of additional ocular comorbidities other than previous clear corneal incision cataract surgery were excluded. Patients from both groups were then matched for age and gender. In cases where both eyes met the inclusion criteria, one eye was randomly chosen in order to avoid biases resulting from intereye correlation [8].

### 2.1. Statistical Analysis

The Student's *t*-test compared the mean and standard deviation for TD-OCT variables in each group. *P* values less than 0.05 were considered statistically significant. A multivariable logistic regression and receiver perating characteristic (ROC) curve analyses representing the area under the curve (AUC) analysis tested the predictive value of the cut-off values to discriminate glaucoma from suprasellar tumors. The statistical power of the current sample to detect a minimal difference of 0.6 mm^2^ in the disc area between the two groups was 98.74%.

## 3. Results

A total of 31 patients met the inclusion criteria and were included in the study: 16 with suprasellar tumors and 15 with POAG. Both groups were similar in terms of gender and age (*P* > 0.05).

### 3.1. Univariate Analysis

Table 1 depicts the mean and standard deviations for the OCT parameters by group.

**Figure 1:**
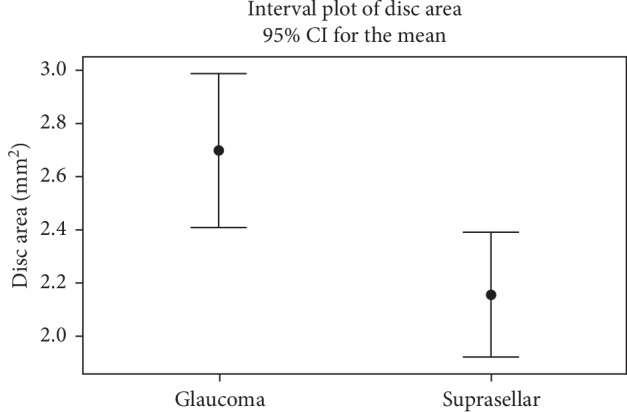
A comparison of the disc area of the glaucoma and suprasellar groups. The dot represents the mean disc area (mm^2^) of each group. The error bars represent the 95% CI of each group.

Briefly, the glaucoma group had a borderline greater total RNFL thickness (74.2 *μ*m versus 62.8 *μ*m, *P*=0.07), greater disc area (2.70 mm^2^ versus 2.16 mm^2^, *P*=0.004), and greater cup volume (0.20 mm^3^ versus 0.08 mm^3^, *P*=0.02).  Figure 1 displays a representation of the difference between the mean disc area (with 95% CI) in the eyes of patients with suprasellar tumors versus eyes of patients with glaucoma.

### 3.2. Multivariate Analysis

Table 2 depicts the results of the multivariate analysis.

Briefly, glaucoma was associated with increased total RNFL thickness (OR = 1.116 per *μ*m, *P*=0.008), increased disc area (OR = 2.402 per 100 *μ*m^2^, *P*=0.002), and decreased rim area (OR = 0.272 per 100 *μ*m^2^, *P*=0.011).

### 3.3. ROC Curve Analysis

**Figure 2:**
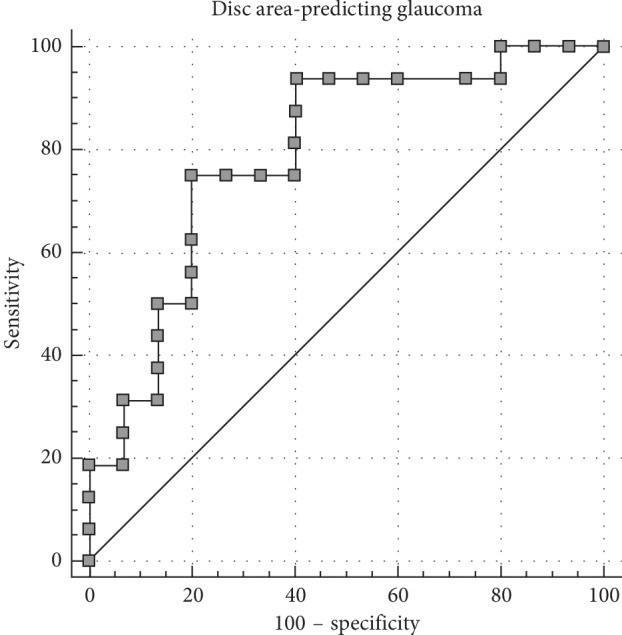
Receiver-operating curve analysis for glaucoma per disc area (mm^2^).

The AUC values of the individual ROC analyses of the TD-OCT and their ability to discriminate glaucoma from suprasellar tumors are depicted in Table 2. The parameter with the greatest AUC that was significant in the multivariate analysis was disc area (Figure 2, AUC = 0.79).

**Figure 3:**
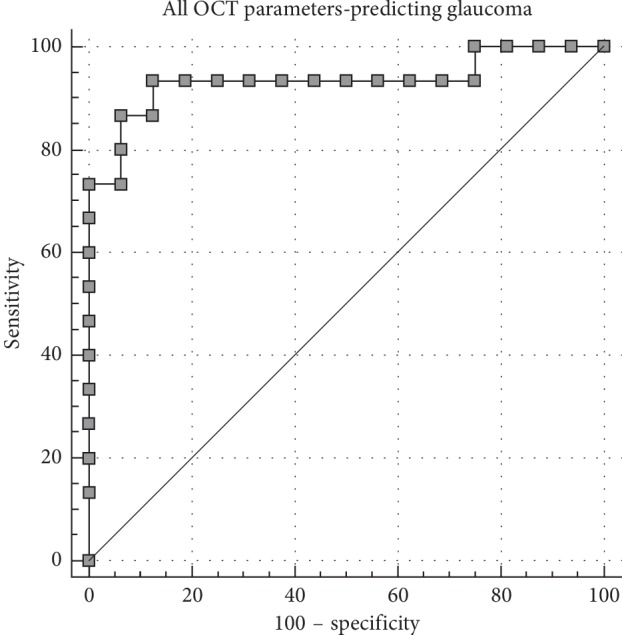
Receiver-operating curve analysis for glaucoma per all TD-OCT parameters combined.

Using the Youden index, the optimal cut-off point identified for stratification was a disc area greater than 2.33 *μ*m^2^. With this cut-off point, there was a sensitivity of 80% and specificity of 75% in stratification. When combining all of the investigated parameters in order to stratify glaucoma from suprasellar tumors, the AUC increased to 0.93 (*P* < 0.001) with a sensitivity of 93.3% and specificity of 87.5% using the Youden index calculated optimal cut-off point (Figure 3).

## 4. Discussion

This study aimed to identify whether disc area and additional TD-OCT parameters could differentiate between suprasellar tumors and open-angle glaucoma in patients with bilateral cupping. Indeed, we found that glaucoma (compared with suprasellar tumors) was associated with increased total RNFL thickness, increased disc area, and decreased rim area with disc area demonstraing the highest AUC of these three.

In the current study, a disc area ≥2.33 mm^2^, measured by TD-OCT, positively predicted glaucoma from suprasellar tumors. Smaller discs were strongly associated with suprasellar tumors, *P*=0.002. Increased disc area was tightly associated with glaucoma, with an odds ratio of 2.40 per each 100 *μ*m^2^. Burgoyne studied a monkey model of glaucoma, revealing that gradual expansion of the optic canal through remodeling of the laminar insertion results in the glaucomatous cup [2]. Canal expansion did not occur in other models of optic neuropathy, such as anterior ischemic optic neuropathy or optic nerve transection [2]. The current study cannot ascertain as to whether there was, in fact, a decrease in the disc area of the suprasellar tumor group (as opposed to an increase in disc area in the glaucoma group). Nevertheless, we believe that given the aforementioned reports on optic canal remodeling, the association between increased disc area and glaucoma in the current study is not a result of susceptibility to glaucoma in patients with large disc areas but rather a result of gradual expansion of the optic canal due to increased intraocular pressures.

In this study, increased total RNFL thickness was associated with glaucoma. In early glaucoma, total RNFL thickness can be expected to differentiate a suprasellar tumor from early glaucoma in cases where overt compressive optic neuropathy occurs. However, in cases of advanced glaucoma, there may be RNFL thinning [9]. As such, the findings of this study regarding a thicker total RNFL thickness in glaucoma (as opposed to suprasellar tumors) do not apply to cases of advanced glaucoma.

This study has several limitations. First of which is its retrospective nature. In addition, the study was based on data from TD-OCT, the leading device used at the time of recruitment. TD-OCT was first introduced to the clinical settings in the 1990s and has evolved towards higher spatial resolution and faster scan speeds [10]. We did not include patients who only had newer SD-OCT scans, since the measurements are not interchangeable in individual cases [11]. The use of SD-OCT to assess neuroretinal rim patterns will, in future studies, offer better morphological analysis and differentiation of Bruch's membrane opening [12, 13]. Indeed, Akashi et al. recently reported that SD-OCT macular analysis can be used to differentiate optic chiasm compression neuropathy from normal eyes. They found that the AUC of the nasal hemiretinal macular RNFL was 0.89 while that of the ganglion cell layer (GCL) combined with the inner plexiform layer (IPL) was 0.988 and that of the GCL combined with RNFL and IPL was 0.981 [14]. An additional limitation of this study is its small sample size; future larger-scale studies may allow the discovery for additional characteristics differentiating between glaucomatous cupping and suprasellar tumors.

In summary, patients with disc cupping are often referred to glaucoma clinics. Larger OCT disc areas, specifically those above 2.33 mm^2^, can serve as an additional sign in early diagnosis of cupping from suprasellar tumors, before compressive visual loss occurs.

## Figures and Tables

**Table 1:** OCT parameters of the suprasellar tumor and glaucoma groups.

Parameter	Craniopharyngioma/pituitary adenoma (*n* = 16)		Glaucoma (*n* = 15)		*P*
	Mean	SD	Mean	SD	
Total RNFL thickness (*μ*m)	62.8	14.7	74.2	18.6	0.07
Disc area (mm^2^)	2.16	0.44	2.70	0.52	0.004
CUP volume (mm^3^)	0.08	0.08	0.20	0.16	0.02
C/D ratio	0.38	0.18	0.49	0.20	0.11
Rim area (mm^2^)	1.38	0.52	1.25	0.56	0.52

RNFL: retinal nerve fiber layer.

**Table 2:** Multivariate analysis of factors predicting glaucoma (as opposed to suprasellar tumor). The area under the curve (AUC) and odds ratio of each factor are presented.

	*P*	AUC	Odds ratio	95% CI	
				Lower	Upper
Total RNFL thickness (per *μ*m)	0.008	0.67	1.116	1.002	1.243
Disc area (per 100 *μ*m^2^)	0.002	0.79	2.402	1.027	5.619
Cup volume (per 100 *μ*m^3^)	0.194	0.82	4.146	0.354	48.595
Cup/disc ratio (per 0.01)	0.071	0.65	0.765	0.551	1.063
Rim area (per 100 *μ*m^2^)	0.011	0.56	0.272	0.070	1.058

RNFL: retinal nerve fiber layer; AUC: area under the curve in individual ROC analyses.

## Data Availability

Data will be made available upon request.
